# Oncocytic Papillary Thyroid Carcinoma and Oncocytic Poorly Differentiated Thyroid Carcinoma: Clinical Features, Uptake, and Response to Radioactive Iodine Therapy, and Outcome

**DOI:** 10.3389/fendo.2021.795184

**Published:** 2021-12-16

**Authors:** Jelena Lukovic, Irina Petrovic, Zijin Liu, Susan M. Armstrong, James D. Brierley, Richard Tsang, Jesse D. Pasternak, Karen Gomez-Hernandez, Amy Liu, Sylvia L. Asa, Ozgur Mete

**Affiliations:** ^1^ Department of Radiation Oncology, University of Toronto, Toronto, ON, Canada; ^2^ Radiation Medicine Program, Princess Margaret Cancer Centre, University Health Network, Toronto, ON, Canada; ^3^ Department of Biostatistics, Princess Margaret Cancer Centre, University Health Network, Toronto, ON, Canada; ^4^ Department of Laboratory Medicine and Pathobiology, University of Toronto, Toronto, ON, Canada; ^5^ Department of Surgical Oncology, University Health Network, Toronto, ON, Canada; ^6^ Endocrine Oncology Site Group, Princess Margaret Cancer Centre, University Health Network, Toronto, ON, Canada; ^7^ Department of Pathology, University Hospitals Cleveland Medical Center, Cleveland, OH, United States; ^8^ Department of Pathology, Case Western Reserve University, Cleveland, OH, United States; ^9^ Department of Pathology, University Health Network, Toronto, ON, Canada

**Keywords:** oncocytic carcinoma, Hürthle cell cancer, oncocytic thyroid carcinoma, radioactive iodine, poorly differentiated thyroid carcinoma

## Abstract

**Objective:**

The main objective of this study was to review the clinicopathologic characteristics and outcome of patients with oncocytic papillary thyroid carcinoma (PTC) and oncocytic poorly differentiated thyroid carcinoma (PDTC). The secondary objective was to evaluate the prevalence and outcomes of RAI use in this population.

**Methods:**

Patients with oncocytic PTC and PDTC who were treated at a quaternary cancer centre between 2002 and 2017 were retrospectively identified from an institutional database. All patients had an expert pathology review to ensure consistent reporting and definition. The cumulative incidence function was used to analyse locoregional failure (LRF) and distant metastasis (DM) rates. Univariable analysis (UVA) was used to assess clinical predictors of outcome.

**Results:**

In total, 263 patients were included (PTC [n=218], PDTC [n=45]) with a median follow up of 4.4 years (range: 0 = 26.7 years). Patients with oncocytic PTC had a 5/10-year incidence of LRF and DM, respectively, of 2.7%/5.6% and 3.4%/4.5%. On UVA, there was an increased risk of DM in PTC tumors with widely invasive growth (HR 17.1; p<0.001), extra-thyroidal extension (HR 24.95; p<0.001), angioinvasion (HR 32.58; p=0.002), focal dedifferentiation (HR 19.57, p<0.001), and focal hobnail cell change (HR 8.67, p=0.042). There was additionally an increased risk of DM seen in male PTC patients (HR 5.5, p=0.03).The use of RAI was more common in patients with larger tumors, angioinvasion, and widely invasive disease. RAI was also used in the management of DM and 43% of patients with oncocytic PTC had RAI-avid metastatic disease. Patients with oncocytic PDTC had a higher rate of 5/10-year incidence of LRF and DM (21.4%/45.4%; 11.4%/40.4%, respectively). Patients with extra-thyroidal extension had an increased risk of DM (HR 5.52, p=0.023) as did those with angioinvasion. Of the patients with oncocytic PDTC who received RAI for the treatment of DM, 40% had RAI-avid disease.

**Conclusion:**

We present a large homogenous cohort of patients with oncocytic PTC and PDTC, with consistent pathologic reporting and definition. Patients with oncocytic PTC have excellent clinical outcomes and similar risk factors for recurrence as their non-oncocytic counterparts (angioinvasion, large tumor size, extra-thyroidal extension, and focal dedifferentiation). Compared with oncocytic PTCs, the adverse biology of oncocytic PDTCs is supported with increased frequency of DM and lower uptake of RAI.

## Introduction

Oncocytic thyroid carcinomas, previously known as “Hürthle cell” carcinomas, are thought to represent a category of thyroid tumors with genetic and clinical characteristics that differ from non-oncocytic thyroid cancers (1–3). It has been suggested that only 10% to 38% of oncocytic thyroid carcinomas take up RAI; resultantly, the use of RAI in this population has been inconsistent (3, 4). Recent evidence suggests that RAI may improve overall survival (OS) in patients with differentiated oncocytic follicular cell thyroid carcinomas (5–7) whereas others argue that there is no evidence to support its use (5, 8, 9). Review of data on “Hürthle cell” carcinomas in the National Cancer Data Base from 1998 to 2006 identified that the use of RAI improved survival at 10 years from 65.0% to 74.4% (p<0.001) (6). One retrospective study showed uptake by metastatic disease in greater than 70% of patients with distant metastasis (DM) at presentation supporting the use of RAI in this patient population (10). These data suggest that there may be an indication for the use of RAI in the management of patients with oncocytic thyroid cancers, although there remains ongoing uncertainty.

Most of the published literature on oncocytic thyroid carcinomas comprises a heterogeneous group of thyroid cancers with oncocytic cytomorphology grouped together as “Hürthle cell” carcinomas. The heterogeneity of these populations of tumors may explain the lack of consistency in RAI response (5, 8, 9). Additionally, the published data do not meticulously distinguish the various types of oncocytic thyroid cancers including follicular variant and classical papillary thyroid carcinomas (PTCs), follicular thyroid carcinoma (oncocytic/Hürthle cell thyroid carcinoma), and poorly differentiated thyroid carcinomas (PDTCs) (11). The scant data on clinicopathologic characteristics of oncocytic PTCs stem from small clinical series (1, 7, 10, 12–14). In some cohorts, patients with oncocytic PTC had more frequent locally invasive growth, extra-thyroidal extension and lymph node metastasis (1, 10, 14). In general, however, oncocytic PTCs were not significantly different from their matched non-oncocytic counterparts with respect to long-term outcome (7, 12). In one clinical series, oncocytic PTCs were not significantly different from oncocytic/Hürthle cell thyroid carcinomas with respect to their overall clinicopathologic variables and favourable RAI response (10).

We postulate that rather than oncocytic cytomorphology and its related molecular alterations, accurate tumor classification and pathological risk factors (e.g. angioinvasion, dedifferentiation) play a central role in the prognosis of these tumors. The Princess Margaret Cancer Center is a large quaternary cancer centre in Toronto, Ontario, Canada where all patients with endocrine malignancies have had their pathology reviewed by experienced endocrine pathologists (SLA, OM) and classified according to a uniform definition and detailed diagnostic workup (11, 15, 16). This unique and rigorous approach has enabled review of clinical and morphologic features, treatment patterns, and outcomes of a consistent disease cohort over time. The main objective of this study was to review the clinicopathologic characteristics and outcome of patients with oncocytic PTC and to compare them with oncocytic PDTC; due to the small number of oncocytic follicular cell thyroid carcinomas, they were not included (17). The secondary objective was to evaluate the prevalence and outcomes of RAI use in this population.

## Materials and Methods

### Study Protocol and Research Ethics Board Approval

This study was initiated following institutional Research Ethics Board approval. It is a retrospective cohort study and the “Strengthening the Reporting of Observational Studies in Epidemiology” (STROBE) statement was used as a guide for reporting of this study (18).

### Source of Data, Participants, and Treatment

Patients with oncocytic PTCs and oncocytic PDTCs who were seen at a quaternary cancer center between 2002 and 2017 were identified from an institutional database and assessed for inclusion. All patients had a pathology review completed by at least one of two experts with consistent reporting and definition (11, 15, 16). In some cases, the patient was referred at the time of recurrence and the pathology review of original material took place during the study period; for those patients, the time of diagnosis was the date of the original surgery and may have ante ceded the onset of the study. The study included only patients who were treated at our institution during the pre-specified period. Therefore, patients who were seen for a second opinion and who received their treatment elsewhere were excluded, due to the unavailability of medical records and follow-up data. In addition, patients with synchronous non-oncocytic thyroid carcinomas with adverse or high-grade features were excluded. Primary treatment included either a total thyroidectomy or staged thyroidectomy. The use of RAI ablation following thyroidectomy was variable and is discussed in the Results Section. External beam radiation therapy (EBRT) was rarely used. Local and regional recurrences were managed surgically where possible. Clinical and pathological data as well as treatment details were retrospectively collected.

### Tumor Classification

**Figure 1:**
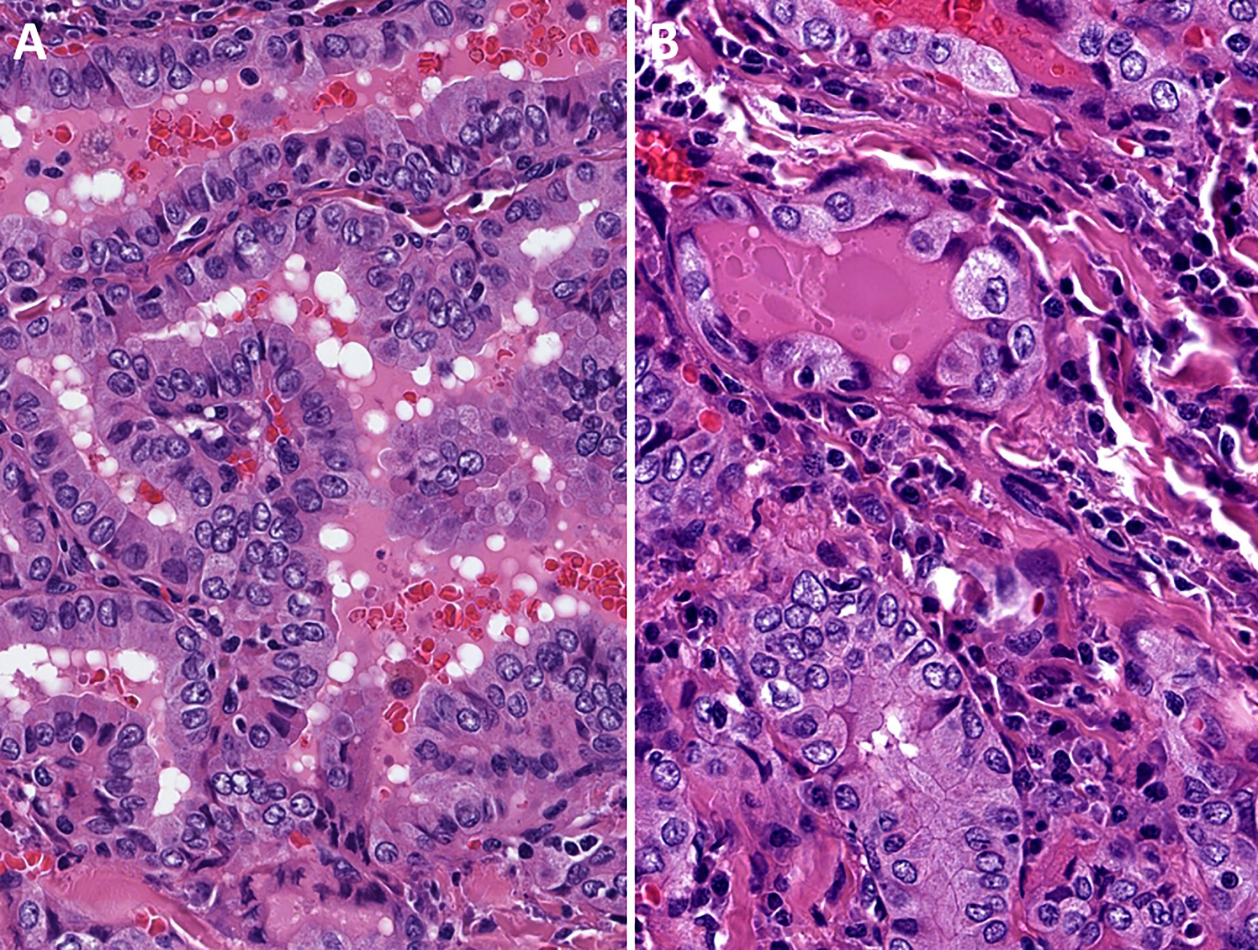
Oncocytic classic variant papillary thyroid carcinoma. These tumors are oncocytic follicular cell-derived neoplasms that exhibit classic (papillary) architecture along with nuclear features of papillary thyroid carcinomas in the absence of tumor necrosis or increased mitotic activity.

**Figure 2:**
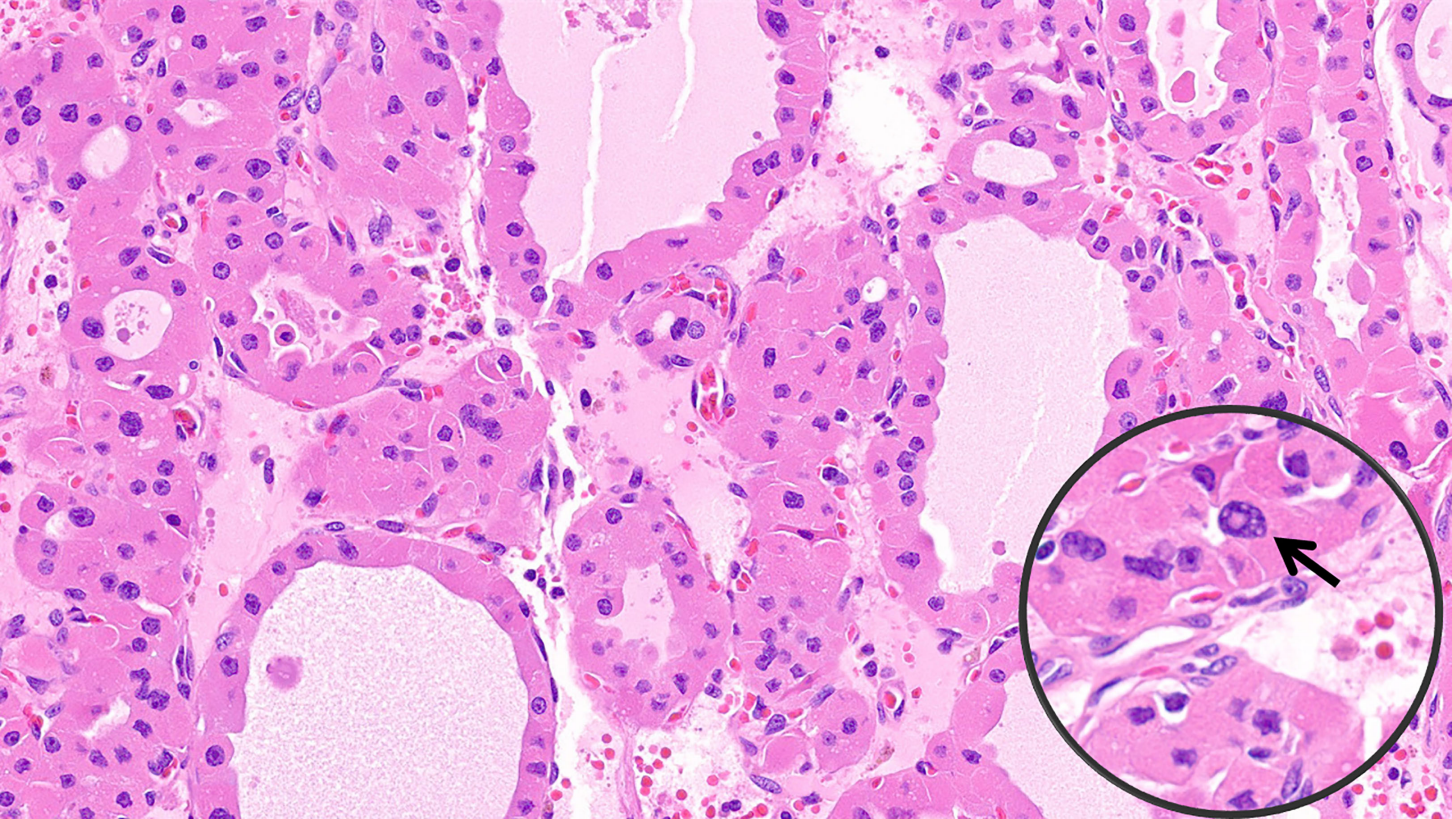
Oncocytic follicular variant papillary thyroid carcinoma. These tumors are oncocytic follicular cell-derived neoplasms that exhibit exclusive follicular architecture along with nuclear features of papillary thyroid carcinomas in the absence of classic architecture, solid growth (>30%), tumor necrosis or increased mitotic activity. The nuclear alterations of papillary thyroid carcinoma are characterized by nuclear membrane irregularities in enlarged nuclei. The inset illustrates an intranuclear pseudoinclusion.

**Figure 3:**
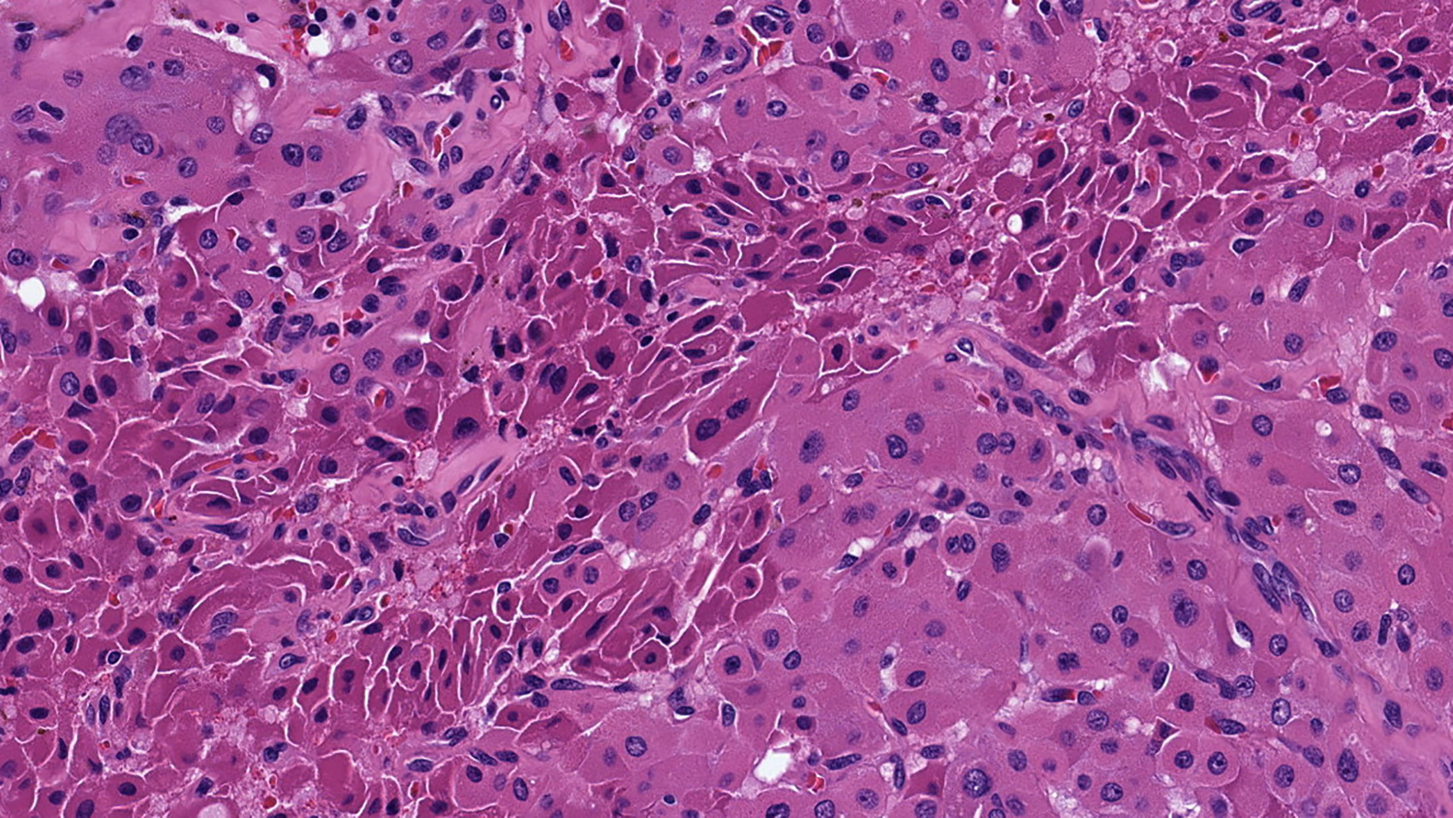
Oncocytic poorly differentiated thyroid carcinoma. These tumors are oncocytic follicular cell-derived neoplasms that show solid/trabecular/insular growth in association with tumor necrosis and/or increased mitotic activity.

• *Oncocytic PTCs* are defined as follicular cell-derived thyroid neoplasms with more than 75% oncocytic change and nuclear features of PTC in the absence of tumor necrosis or increased mitotic activity (>3 per 2mm^2^). These tumors are further subtyped based on their growth patterns into oncocytic classical (**Figure 1**) and oncocytic follicular variants (**Figure 2**).
• *Oncocytic PDTCs* are defined as invasive follicular cell-derived thyroid neoplasms with more than 75% oncocytic change that have solid/trabecular/insular growth in association with increased mitotic activity (>3 per 2mm^2^) and/or necrosis **(** **Figure 3**) (11). In general, these tumors do not have florid nuclear features of PTC.
• *Oncocytic PTCs with focal dedifferentiation* are defined as oncocytic PTCs that contain a poorly differentiated thyroid carcinoma component that accounts for less than 30% of the tumor volume.
• *Extra-thyroidal extension* is defined as tumor invasion into the strap muscle or beyond (19). Invasion of perithyroidal fibroadipose soft tissue is not classified as extra-thyroidal extension (20).
• *Vascular invasion* is defined as tumor invading through the wall of a blood vessel and/or intravascular tumor cells, either one admixed with thrombus (21). Tumor cells in lymphatic channels is distinguished as lymphatic invasion and is not classified as vascular invasion.
• *Widely invasive* carcinomas are defined as tumors in which tumor invasion was seen grossly or was associated with extensive intrathyroidal or extra-thyroidal tumor spread.

### Definition of Outcome, Data Sources, and Measurement

Locoregional failure (LRF) times were defined from the date of treatment completion to the date of local and/or regional recurrence. This was defined by physical examination or radiographic imaging, whichever came first. Time of diagnosis of distant metastases (DM) was defined from the date of diagnosis to date of distant disease identification. DM was based on follow-up radiographic imaging; this was performed on the basis of rising thyroglobulin, new indicative symptoms, or additional imaging following the diagnosis of local and/or regional failure. Biopsies were obtained in the setting of DM only if there was a concern about a different diagnosis. All data were retrospectively abstracted from paper and electronic medical records. Data were recorded similarly for all patients treated at our institution over time. There was no blinding in the assessment of the outcome. Overall survival (OS) was calculated from the date of diagnosis to the date of last follow-up or death. Progression-free survival (PFS) was calculated from the date of diagnosis to the date of any outcome (LRF or DM) or death, censored at the date of last follow-up.

### Study Size, Endpoints, and Statistical Data Analysis

The total study size was determined by including all patients who met inclusion criteria for oncocytic PTC and oncocytic PDTC during the study period. Descriptive statistics were used for patient demographics and treatment details. Patients with oncocytic PTC (e.g., oncocytic classic variant PTC, oncocytic follicular variant PTC) and oncocytic PDTC were analyzed separately. The cumulative incidence function with death as competing risk was used to analyze local failure (LF), regional failure (RF) and distant metastasis (DM) rates for this cohort of patients. Univariable Fine and Gray competing risk regression models were fitted to assess clinical predictors of outcome for patients with oncocytic PTC and oncocytic PDTC. Variables with unknown values were treated as missing and no imputation for missing data was used. The Kaplan-Meier method was used to estimate OS and PFS. P values <0.05 were considered statistically significant. Due to the low number of events, multivariate analyses were not able to be performed. All analyses were conducted using R version 4.0.2 (22).

## Results

### Whole Cohort Patient Demographics and Follow-Up

**Figure 4:**
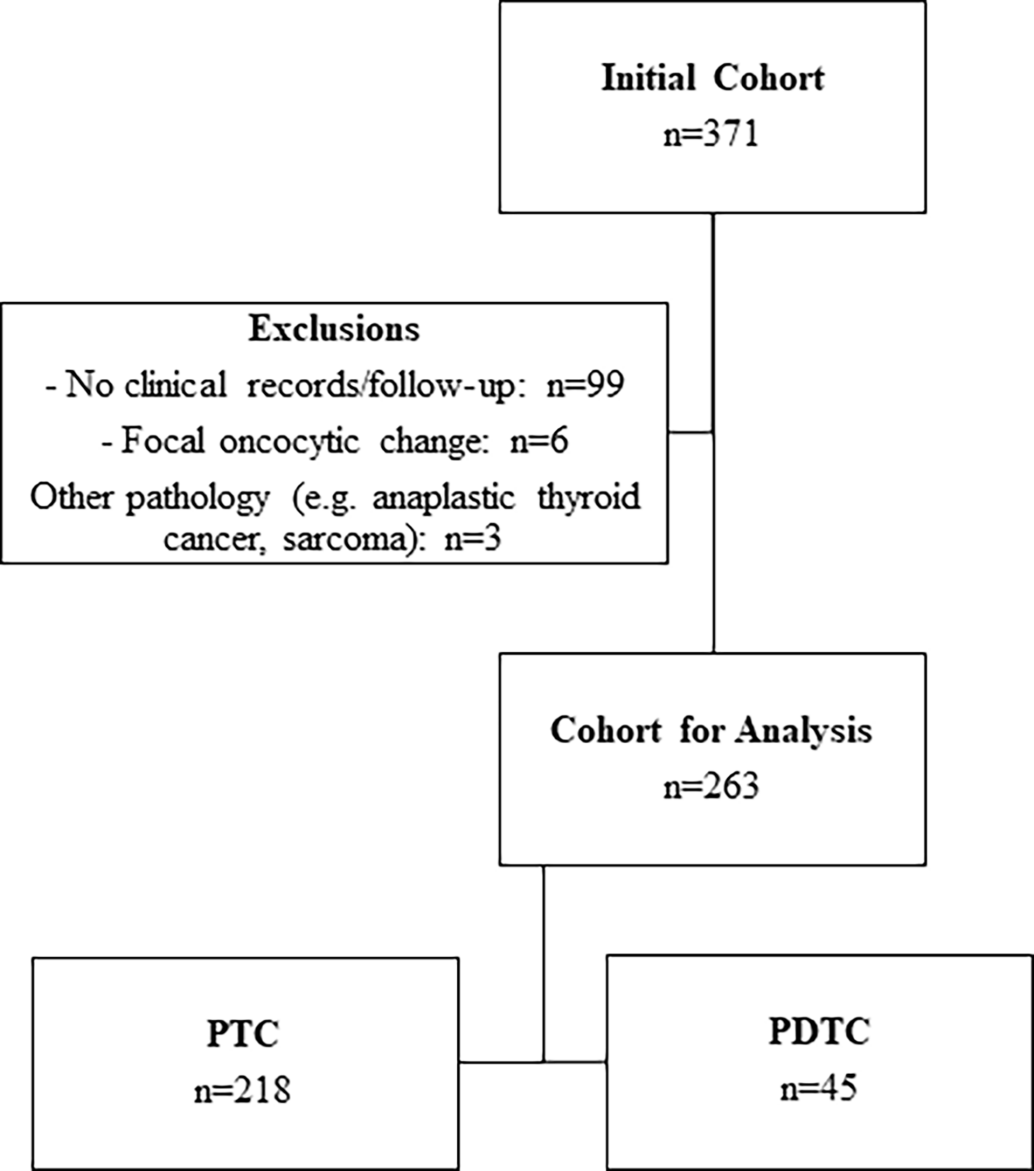
Consort diagram.

In total, 371 cases of oncocytic PTC and oncocytic PDTC were identified from an institutional database. Of these, 108 patients were excluded from analyses due to lack of available records or follow-up data (e.g., treatment and/or follow-up was completed at a different institution; n=99), other significant tumor pathology (n=3), or less than 75% oncocytic change (n=6). In total, 263 patients were included in the final analysis (**Figure 4**). Baseline patient and treatment characteristics are summarized in **Table 1**. Of the 263 patients, 218 had oncocytic PTC (76 oncocytic classic variant PTC, 142 oncocytic follicular variant PTC) and 45 had oncocytic PDTC. The median follow-up time was 4.4 years (range: 0-26.7 years) for the whole cohort, and more specifically it was 4.2 years (range: 0-26.7 years) for patients with oncocytic PTC and 5.1 years (range: 0.2-15.7 years) for patients with oncocytic PDTC.

**Table 1:** Baseline patient and treatment characteristics.

Variable	Oncocytic Papillary Thyroid Carcinoma N = 218 (%)			Oncocytic Poorly Differentiated Thyroid Carcinoma N = 45 (%)
	Whole Cohort N = 218 (%)	Oncocytic follicular variant PTC N = 142 (%)	Oncocytic classic variant PTC N = 76 (%)	
Median follow up (years)	4.2 (0.0-26.7)	3.9 (0.0-26.7)	5.4 (0.3-17.0)	5.1 (0.2-15.7)
Sex				
Female	170 (78)	115 (81)	55 (72)	21 (47)
Male	48 (22)	27 (19)	21 (28)	24 (53)
Median age: years (range)	55.3 (19.4,85)	56.4 (21.0-85.0)	50.2 (19.4-77.8)	63.6 (35.3,83.6)
Age				
≤55	108 (50)	64 (45)	44 (58)	16 (36)
>55	110 (50)	78 (55)	32 (42)	29 (64)
Prior radiation exposure	14 (7)	9 (7)	5 (7)	4 (14)
Type of surgery				
Total thyroidectomy	76 (35)	41 (29)	35 (46)	21 (47)
Staged thyroidectomy	94 (43)	60 (42)	34 (45)	24 (53)
Hemithyroidectomy	48 (22)	41 (29)	7 (9)	
RAI Treatment	112 (51)	60 (42)	52 (68)	39 (86)
**Tumor Characteristics**				
Dominant Lesion >4cm	52 (24)	35 (25)	17 (22)	29 (64)
Multifocal	135 (62)	95 (67)	40 (53)	19 (43)
Widely invasive growth	31 (14)	19 (13)	12 (17)	34 (76)
Extrathyroidal Extension	3 (1)	3 (2)	0 (0)	6 (14)
Positive Margin	15 (7)	10 (7)	5 (7)	15 (34)
Perineural Invasion	4(2)	1 (1)	3 (7)	4(9)
Lymphatic Invasion	16 (7)	4 (3)	12 (16)	11 (25)
Angioinvasion	36 (17)	23 (16)	13 (18)	39 (89)
**Adverse Pathologic Features**				
Focal Dedifferentiation	10 (5)	2	8	n/a
Focal Tall Cell Change	15 (7)	5	10
Focal Hobnail Cell Change	5 (2)	2	3

n/a, not applicable.

### Oncocytic Papillary Thyroid Carcinoma

#### Patient Demographics and Pathologic Features

The median age in this cohort of 218 patients was 55.3 years (range: 19.4 to 85 years) and there was a predominance of female patients (78%). Primary treatment was total thyroidectomy [primary total thyroidectomy: n=76 (35%); staged total thyroidectomy: n=94 (43%)]. Forty-eight patients (22%) had a hemithyroidectomy only (41 – follicular variant PTC and 7 classic variant PTC). In total, 112 (51%) patients received postoperative RAI. Overall, 52 patients (24%) had a dominant lesion >4cm and 31 patients (14%) had widely invasive tumors. With regards to adverse histologic features, ten patients (5%) had focal dedifferentiation (median 10%; range 5%-25%), 15 patients (7%) had focal tall cell change, and 5 patients (2%) had focal hobnail cell change. The pathologic features at baseline for the whole cohort as well as for the subgroups of patients with oncocytic follicular variant and oncocytic classic variant PTC are summarized in **Table 1**.

#### Cumulative Incidence of and Risk Factors for Locoregional or Distant Recurrence

**Figure 5:**
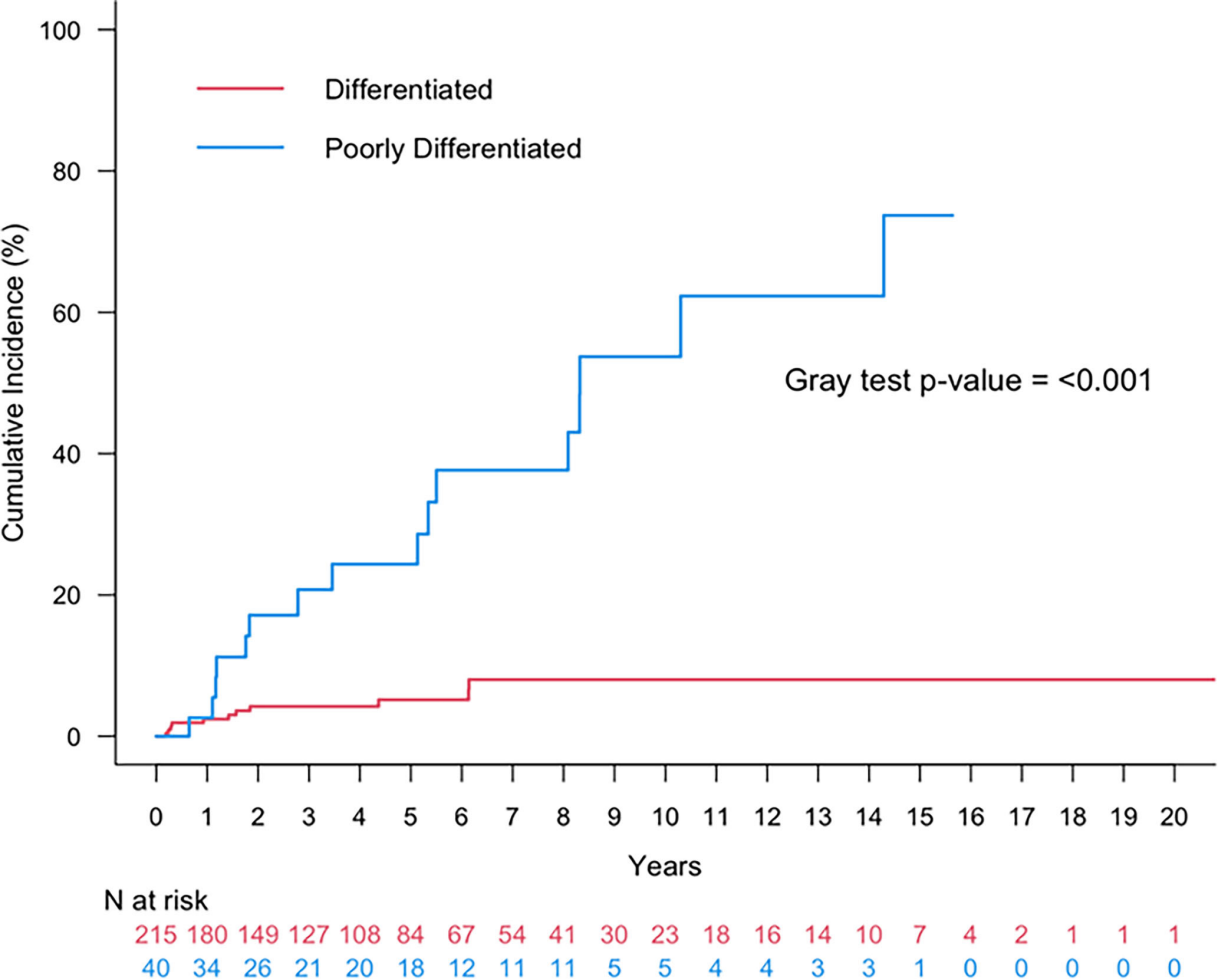
Cumulative incidence of any recurrence (local, regional, distant) or death for patients with oncocytic papillary thyroid carcinoma (red) or oncocytic poorly differentiated thyroid carcinoma (blue).

At the end of the study follow-up, 11 patients (5%) developed a locoregional recurrence. The corresponding 5- and 10-year cumulative incidence of LRF was 2.7% (95% CI: 0.9-6.5%) and 5.6% (95%CI: 2.1-11.8%) (**Figure 5**). **Table 2** shows the univariate analysis of factors associated with LRF. Pathologic factors associated with an increased risk of LR include: widely invasive tumor growth (HR 8.59, p=0.007), extrathyroidal extension (HR 37.15; p<0.001), and focal dedifferentiation (HR 18.18, p<0.001). On subgroup analysis, this appears to be primarily driven by patients with oncocytic classic variant PTC rather than follicular variant PTC. Male patients additionally had an increased risk of LRF (HR 4.86, p=0.03).

**Table 2:** Univariate analysis of factors associated with locoregional recurrence in patients with oncocytic classic variant and oncocytic follicular variant papillary thyroid carcinoma (PTC).

	Whole Cohort		Oncocytic Classic Variant PTC		Oncocytic Follicular Variant PTC	
	HR	P value	HR	P value	HR	P value
**Age >55**	0.59 (0.11-3.09)	0.54	**	0.19	1.66 (0.15,18.1)	0.68
**Male**	4.86 (1.12-21.08)	**0.03**	1.67 (0.18-15.88)	0.65	11.66 (1.32,103.07)	**0.027**
**Tumor size >4cm**	3.29 (0.67-16.23)	0.14	7.82 (0.73-83.91)	0.09	1.43 (0.13,15.99)	0.77
**Widely invasive growth**	8.59 (1.82-40.61)	**0.007**	19.83 (2.28-172.65)	**0.007**	3.74 (0.36,38.66)	0.27
**Extrathyroidal Extension**	37.15 (10.33-133.60)	**<0.001**	15.64 (3.55-68.86)	**<0.001**	74.62 (12.84,433.65)	<**0.001**
**Angioinvasion**	3.85 (0.7-21.26)	0.12	5.36 (0.40-71.11)	0.20	2.72 (0.26,28.19)	0.4
**Focal Dedifferentiation**	18.18 (4.38-75.43)	**<0.001**	45.75 (6.84-306.22)	**<0.001**	**	1.0
**Focal Tall Cell Change**	3.85 (0.55-27.01)	0.17	5.8 (0.55-60.69)	0.14	**	0.83
**Focal Hobnail Cell Change**	**	0.76	**	0.86	not applicable	not applicable

**HR cannot be estimated for these categories as there were no patients with each relevant prognostic factor who developed a local recurrence; the p-value from Gray’s test is reported.

¥There were no patients with oncocytic follicular variant PTC who had focal hobnail cell change in this cohort.

The hazard ratio (HR) along with the 95% confidence interval is reported. Significant p values are highlighted in bold.

Seven patients presented with DM and two additional patients developed DM during the study follow up; the latest recurrence occurred 5.24 years after the initial diagnosis. Four of these patients had oncocytic classic variant PTC and five had oncocytic follicular variant PTC. The 5- and 10-year cumulative incidence of DM was 3.4% (95%CI: 1.3-7.0%) and 4.5% (95%CI: 1.8-9.0%) (**Figure 5**). The univariate analysis of factors associated with increased risk of DM is shown in **Table 3**. Pathologic factors associated with an increased risk of DM included: widely invasive tumor growth (HR 17.1; p<0.001), extrathyroidal extension (HR 24.95; p<0.001), and angioinvasion (HR 32.58; p=0.002). Notably, all patients who developed DM had tumors larger than 4 cm. Male patients additionally had an increased risk of DM (HR 5.5; p=0.03). While almost all patients with DM had angioinvasion, in terms of histology, there was an increased risk of DM in patients with focal dedifferentiation (HR 19.57, p<0.001) and the presence of focal hobnail cell change (HR 8.67, p=0.042). The presence of focal tall cell change did not increase the risk of LRF or DM in this cohort overall. The prognostic factors in the subgroup of patients with oncocytic classic variant PTC and oncocytic follicular variant PTC are also summarized in **Table 3**; caution should be undertaken when interpreting the data given the small numbers of events.

**Table 3:** Univariate analysis of factors associated with distant metastases in patients with oncocytic classic variant and oncocytic follicular variant papillary thyroid carcinoma.

	Whole Cohort		Oncocytic Classic Variant PTC		Oncocytic Follicular Variant PTC	
	HR	P value	HR	P value	HR	P value
**Age >55**	1.43 (0.33-6.18)	0.63	0.79 (0.08-7.71)	0.84	2.53 (0.27,24.07)	0.42
**Male**	5.5 (1.18-25.7)	**0.03**	5.96 (0.52-68.09)	0.15	5.04 (0.67,37.76)	0.12
**Tumor size >4cm**	**	**<0.001**	**	**<0.001**	**	**<0.001**
**Widely invasive growth**	17.7 (3.21-97.56)	**<0.001**	13.14 (1.01-171.65)	**0.049**	21.74 (2.22,213.28)	**0.008**
**Extrathyroidal Extension**	24.98 (4.11-151.94)	**<0.001**	15.27 (1.44-161.43)	**0.023**	40.16 (3.25,496.56)	**0.004**
**Angioinvasion**	32.58 (3.71-286.21)	**0.002**	9.66 (0.83-111.74)	0.07	****	**<0.001**
**Focal Dedifferentiation**	19.57 (4.77-80.23)	**<0.001**	19.76 (2.26-172.51)	**0.007**	65.83 (16.56,261.72)	<**0.001**
**Focal Tall Cell Change**	2.57 (0.3-21.98)	0.39	4.16 (0.4-43.32)	0.23		0.71
**Focal Hobnail Cell Change**	8.67 (1.08-69.72)	**0.042**	28.14 (1.99-398.59)	**0.014**		**0.8**

**HR cannot be estimated. All patients with distant metastases had a dominant oncocytic papillary thyroid carcinoma greater than 4 cm. The p-value from Gray’s test was reported.

****All patients with oncocytic follicular variant PTC who developed distant metastases had angioinvasion.

The hazard ratio along with the 95% confidence interval is reported. Significant p values are highlighted in bold.

Finally, the 5- and 10-year cumulative incidence of any recurrence (local, regional, or distant) was 5.2% (95%CI: 2.5-9.3%) and 8.0% (95%CI: 3.9-14.1%).

#### Use of Radioactive Iodine

In the group of 218 patients with oncocytic PTCs, larger tumour size (> 4cm), widely invasive disease, and angioinvasion were associated with the use of postoperative RAI. Cox proportional hazards model was performed for OS and PFS. There was no difference in OS (HR 0.71, 95% CI: 0.1-5.06; p=0.73) for patients who received RAI compared with those who did not. Similarly, there was no difference in PFS for patients who received RAI (HR1.95; 95% CI: 0.61-6.26; p=0.26).

The patients who presented with DM (lung n=2, bone n=4, lung + bone n=1) were initially treated with total thyroidectomy and postoperative RAI. Only three of 7 (42.8%) patients had metastatic disease that showed RAI uptake; two patients had focal dedifferentiation on their initial pathology. Two additional patients developed DM during the study follow up time and were treated with palliative EBRT.

### Oncocytic Poorly Differentiated Thyroid Carcinomas

#### Patient Demographics

In total, 45 patients with oncocytic PDTC were included in the analysis. There was a slight predominance of male patients (53%). The median age of presentation was older than for patients with oncocytic PTC (63.6 years, range: 35.5-83.6 years). RAI use was frequent and used in the management of 39 patients (86%). Four patients (8%) received EBRT alone or in addition to RAI.

#### Cumulative Incidence of and Risk Factors for Locoregional or Distant Recurrence

The 5- and 10-year cumulative incidence of LRF was 21.40% (95% CI: 9.1-37.1%) and 45.4% (95%CI: 24.2-64.4%). Within the cohort of patients with oncocytic PDTCs, 16 (36%) had distant metastatic disease (DM). The corresponding 5- and 10-year cumulative incidence of DM was 11.4% (95%CI: 3.5-24.4%) and 40.4% (95%CI: 17.5-62.4%). The 5- and 10-year cumulative incidence of any recurrence (local, regional, or distant) was 24.4% (95%CI: 11.1-40.3%) and 53.7% (95%CI: 30.5-72.2%) (**Figure 5**).

**Table 4** shows the univariate analysis of factors associated recurrence. There were no classic demographic (e.g., age >55, sex) or pathologic factors (e.g., tumor size >4cm, extrathyroidal extension, etc.) associated with an increased risk of LRF. Conversely, all patients who developed DM had evidence of angioinvasion. Additionally, patients with extrathyroidal extension more frequently developed DM with respective HR: 5.52; p=0.032 (**Table 4**).

**Table 4:** Univariate analysis of factors associated with locoregional recurrence or distant recurrence in patients with oncocytic poorly differentiated thyroid carcinoma.

	Locoregional Recurrence HR	P value	Distant Metastases HR	P value
**Age >55**	1.76 (0.64-4.81)	0.27	0.87 (0.28-2.73)	0.81
**Male**	0.53 (0.17-1.65)	0.28	1.29 (0.4-4.11)	0.67
**Tumor size >4cm**	0.44 (0.15-1.27)	0.13	0.49 (0.17-1.42)	0.19
**Widely invasive growth**	0.87 (0.24-3.15)	0.84	1.11 (0.24-5.17)	0.89
**Extrathyroidal Extension**	1.39 (0.36-5.39)	0.63	5.52 (1.15-26.38)	**0.03**
**Angioinvasion**	0.63 (0.05-7.35)	0.72	**	**<0.001**

**HR cannot be estimated. All patients with distant metastases had angioinvasion. The p-value from Gray’s test was reported. Significant p values are highlighted in bold.

#### Use of RAI Incidence and Management of Distant Metastases in Patients With Oncocytic PDTC

The demographics, pathologic features, and management of DM are summarized in **Table 5**. All 16 patients with DM had widely invasive tumors and angioinvasive carcinoma. Seven patients presented with metastatic disease at the time of diagnosis in the lungs (n=2), bone (n=2), bone + lungs (n=2), and lungs + bones + liver (n=1). All patients were treated with a total thyroidectomy followed by postoperative RAI (median dose = 150mCi, range: 100-200mCi); only three patients had DM that demonstrated RAI uptake. Nine additional patients developed metastatic disease during the study follow-up at a median time of 5.51 years (range: 1.17 – 10.31 years). Three of these patients received RAI and only one had disease that demonstrated RAI uptake. Therefore, in total, four patients with PDTC and DM out of 10 who received RAI (40%) had RAI-avid disease.

**Table 5:** Incidence, characteristics, and management of distant metastases in patients with oncocytic papillary thyroid carcinoma and oncocytic poorly differentiated thyroid carcinoma.

Demographics	Primary Treatment	Pathology	Timing DM Diagnosis (years)	DM location	DM Management
**Oncocytic PTC**					
54y male	TT + neck dissection + RAI 100mCi	8cm oncocytic classic variant PTC, 10% dedifferentiation, widely invasive, angioinvasive 5/36+LNs	5.24	Lung, Bone	Palliative EBRT to bone met
65y male	TT + RAI 125mCi	7.2cm oncocytic follicular variant PTC, minimal capsular invasion, angioinvasive	Diagnosis	Bone	Surveillance – slow growth over time, asymptomatic
48y female	TT + RAI 200mCi	2.5cm oncocytic follicular PTC, 10% dedifferentiation, widely invasive, angioinvasive	Diagnosis	Bone	RAI 200mCi – initial uptake; repeat RAI no further uptake – offered EBRT
65y male	TT + RAI 125mCi	7.5cm oncocytic follicular variant PTC, widely invasive, angioinvasive	4.52	Lung, Liver	Palliative EBRT, VEGF offered but declined by patient
53y female	TT + RAI 150mCi + EBRT 60Gy in 30 fractions	Oncocytic follicular variant PTC*, 25% dedifferentiation, widely invasive, angioinvasive, ETE, 3/6+ LNs (level 3)	Diagnosis	Bone	RAI 150mCi – no uptake; palliative EBRT
78y female	TT + RAI 150mCi	6.7cm oncocytic follicular variant PTC, widely invasive, angioinvasive	Diagnosis	Bone, Lung	RAI 150mCi – uptake; considered for further treatment but passed away from other causes
19y male	TT + neck dissection + RAI 150 mCi	6cm oncocytic classic variant PTC, focal 10% tall cell change, focal 5% hobnail cell change, widely invasive, angioinvasive, perineural invasion, 27/51+LNs (level 2-5)	Diagnosis	Lungs	RAI 150mCi – no uptake; ongoing surveillance
49y male	TT + RAI 150mCi	0.9cm oncocytic classic variant PTC (papillary microcarcinoma), locally invasive	Diagnosis	Bone	RAI 150mCi – no uptake; resection of bone met + postoperative RT
74y female	TT + RAI 100mCi	5.8cm oncocytic classic variant PTC, 10% dedifferentiation	Diagnosis	Lungs	RAI 100mCi – uptake
**Oncocytic PDTC**					
65y male	TT + RAI 150mCi	6cm oncocytic PDTC, widely invasive, angioinvasive, ETE	1.5	Lung, Bone	Palliative due to poor performance status
35y female	TT + central neck dissection + RAI 150mCi	5.7cm oncocytic PDTC, widely invasive, ETE, angioinvasive	Diagnosis	Lung	RAI 150mCi – uptake at all sites of metastatic disease; repeat RAI (200mCi, no further uptake)
78y female	TT + RAI 200mCi	2cm oncocytic PDTC, widely invasive, angioinvasive	Diagnosis	Lung, Bone	RAI 200mCi – no uptake; VEGF
45y female	ST + RAI 125mCi	3cm oncocytic PDTC, widely invasive, angioinvasive, ETE,	5.35	Lung	Surveillance
51y male	ST + RAI 100mCi	6.5cm oncocytic PDTC, minimally invasive, extensive angioinvasion	8.32	Lung	RAI 150mCi – no uptake; VEGF
75y female	TT + 100mCi	4cm oncocytic PDTC, widely invasive, angioinvasive	7.87	Lung	EBRT (palliative due to poor performance status)
70y male	TT + RAI 150mCi	4cm oncocytic PDTC, minimally invasive, angioinvasive	Diagnosis	Bone	RAI 150mCi – no uptake; resection of bone met + postoperative EBRT
46y female	ST + RAI 100mCi	4cm oncocytic PDTC, widely invasive, extensive angioinvasion, 0/2 LNs	9.14	Bone	SBRT
51y male	TT + RAI 125mCi	7cm oncocytic PDTC, widely invasive, angioinvasive	1.17	Lung	RAI 125mCi – no uptake; resection
67y male	TT + neck dissection + RAI 100mCi	3cm oncocytic PDTC, widely invasive, angioinvasive, 2/27+LNs (level 2)	Diagnosis	Lung, Bone, Liver	RAI 100mCi – uptake at all site of metastatic disease
58y male	HT + EBRT 66Gy in 33 fractions (unresectable disease)	4cm oncocytic PDTC, widely invasive, angioinvasive	5.51	Lung	SBRT
46y female	TT + RAI 200mCi	1.9cm oncocytic PDTC, widely invasive, angioinvasive, ETE, perineural invasion	Diagnosis	Bone	RAI 200mCi – mild uptake; resection + postoperative EBRT (50Gy in 20 fractions); VEGF
72y female	TT + neck dissection + EBRT 66Gy in 33 fractions + RAI 150mCi	8.2cm oncocytic PDTC, widely invasive, angioinvasive 11/48+ LNs (5cm, + ENE, level 2)	Diagnosis	Lung, Bone	RAI 150mCi – no uptake; palliative EBRT
60y female	ST + neck dissection + RAI 150mCi	1.3cm oncocytic PDTC, widely invasive, angioinvasive, ETE, 0/20 LNs	Diagnosis	Lung	RAI 150mCi – no uptake; VEGF
64y male	TT + EBRT 66Gy in 33 fractions + RAI 150mCi	5.6cm oncocytic PDTC, widely invasive, angioinvasive	1.83	Lung	VEGF
37y male	TT + RAI 200mCi	9.5cm oncocytic PDTC, widely invasive, angioinvasive, perineural invasion	10.31	Lung	RAI 200mCi – uptake; VEGF

PTC, Papillary thyroid carcinoma; PDTC, Poorly differentiated thyroid carcinoma; TT, total thyroidectomy; ST, staged thyroidectomy; HT, hemithyroidectomy; EBRT, external beam radiation therapy; SBRT, stereotactic body radiation therapy; RAI, radioactive iodine; ETE, extrathyroidal extension.

^α^No other adverse features including no widely invasive disease and no angioinvasion.

*The accurate tumor size could not be determined; the listed pathology data is generated from the thyroid bed/central neck recurrence.

## Discussion

Oncocytic PTCs are part of the differentiated thyroid carcinoma spectrum and there remains significant controversy about their biological behavior and optimal management. We reviewed the management and outcomes of a cohort of patients with oncocytic PTCs and PDTCs as defined in a uniform manner according to specific pathological criteria.

A wide range of outcomes has been reported for patients with oncocytic PTC, likely secondary to the lack of distinction between various types of oncocytic PTC and heterogenous grouping into “Hürthle cell” thyroid carcinomas. Some authors report that patients with oncocytic PTCs generally have an excellent prognosis with reported 5- and 10-year disease-free survival of 93% and 81%, respectively, and less than 10% of patients developing local or distant recurrence (7, 14). Similar results were seen in our study with a cumulative incidence of any recurrence (local, regional, or distant) at 10 years of 8.0% (95%CI: 3.9-14.1%). Conversely, a retrospective study from the Mayo Clinic described 5- and 10-year rates of locoregional or distant recurrence of 11 and 28%, respectively (23). The poorer outcomes seen in the Mayo Clinic study are likely due to changes in treatment guidelines during the 32-year study period as well as inconsistencies and changes to the histologic criteria for the diagnosis of various forms of thyroid carcinomas with oncocytic change.

RAI use in the setting of oncocytic PTC is variable due to the suggestion that there is limited uptake of RAI and therefore limited benefit. Approximately half of the patients with oncocytic PTC received RAI in our cohort – the clinical factors predictive of RAI use were similar to non-oncocytic PTCs and included: large lesions (>4cm), widely invasive disease, and angioinvasion. This is similar to previous reports that suggest that RAI is more commonly used in the setting of larger tumors, extrathyroidal extension, and presence of distant metastases (5, 24). There was no difference in OS nor PFS in the group of patients with differentiated oncocytic PTC based on receipt of RAI; the number of events was too small to perform additional subgroup analyses. This is similar to the observed behaviour of non-oncocytic PTC, with published data suggesting no benefit to RAI in patients with low risk disease (25–27).

In our study, nine patients with oncocytic PTC had DM (seven at diagnosis and two during study follow-up). Among these, four had also focal dedifferentiation characterized by a focal tumor component (less than 30% of the tumor volume) with solid growth, increased mitotic activity and/or necrosis. Focal dedifferentiation has been shown to alter the prognosis of patients with PTCs in former series (28, 29). However, the current series also underscored that this finding also correlates with adverse biology. RAI was used in the seven patients who presented with DM and only three (42.8%) had metastatic disease demonstrating uptake. This number is lower than the reported uptake in patients with metastatic differentiated non-oncocytic PTC, with RAI uptake reported in up to two thirds of these patients (30, 31). Interestingly, of the three patients with RAI uptake two had evidence of focal dedifferentiation. Overall, the total number of events in our cohort was too low to perform additional exploratory analyses of factors predicting RAI avidity during the course of follow-up time.

Compared with patients with oncocytic PTCs, patients with oncocytic PDTCs were older, more likely to be male and had worse outcomes in terms of any recurrence (5-year and 10-year cumulative incidence of any recurrence was 24.4% and 53.7%, respectively). Two additional retrospective studies confirm these results and suggest oncocytic PDTCs follow a more aggressive course (32, 33). Bai et al. found that oncocytic PDTC patients had higher tumour-specific mortality and exhibited more extensive vascular invasion (angioinvasion) than other differentiated oncocytic cell thyroid carcinoma patients. At final follow-up, ranging from 4 to 120 months, 17% of patients had developed a recurrence and 83% had distant metastases (32). Papotti et al. had similar findings—oncocytic PDTCs were more aggressive than stage-matched well-differentiated oncocytic follicular cell thyroid carcinomas (33). In their study, 37% of patients had local recurrences or distant metastases at last follow-up. Skeletal muscle infiltration and extensive vascular invasion were markers of poor prognosis in that series (33). In our series, metastases were more common in patients with oncocytic PDTCs. Patients with DM at the time of diagnosis were treated with a total thyroidectomy and post-operative RAI to a median dose of 150mCi (range: 100-200mCi). The management of metastases that developed during the study follow-up was variable and included surveillance, RAI, EBRT, or systemic therapy. Of the patients who received RAI, less than 50% have had RAI-avid metastatic disease.

The main limitations of the present study are those inherent to its retrospective cohort design and relatively short follow-up time (median 4.4 years). Although 5- and 10-year outcome data are reported, there are relatively small numbers of patients remaining in the cohort at these times and thus these numbers should be interpreted with caution. The findings, however, are strengthened by both the sample size and the rigorous and consistent endocrine pathology workup throughout the study period. The total number of events is small in the cohort of differentiated thyroid carcinoma patients for in depth statistical analyses of predictors of RAI-avidity and outcome. In addition, the small number of events in the context of small numbers of certain prognostic factors in this cohort (e.g. extra-thyroidal extension) led to very high hazard ratios and corresponding confidence intervals; these should be interpreted with caution. This, however, implies that this well-defined oncocytic PTC cohort has excellent clinical outcomes and oncocytic PTCs may be managed similarly to their non-oncocytic counterparts. Within the limitation of the small number of patients with metastatic disease, it may appear as though oncocytic PTCs have reduced RAI uptake in the setting of DM compared with their non-oncocytic counterparts. Given that we do not have a good way of predicting which patients in this cohort may benefit from RAI and that the side-effect profile of RAI is favorable, it may be a consideration for patients with high risk features after careful discussion.

In conclusion, we present a large cohort of patients with oncocytic PTC and oncocytic PDTC. We demonstrated that patients with oncocytic PTC have excellent clinical outcomes and low rates of recurrence. Similar to their non-oncocytic counterparts, the status of angioinvasion, large tumor size (>4 cm), extrathyroidal extension, and focal dedifferentiation stand out as dynamic risk factors for patients with oncocytic PTCs. Compared to their non-oncocytic counterparts, however, it appears that a smaller proportion of patients who develop metastatic disease demonstrate RAI uptake; although the absolute numbers of patients in our cohort are small. Finally, compared with differentiated PTCs, the adverse biology of oncocytic PDTCs is also supported with increased frequency of distant metastases and decreased survival. Further prospective studies are required to better define the optimal use of RAI in this population.

## Data Availability Statement

The original contributions presented in the study are included in the article/supplementary material. Further inquiries can be directed to the corresponding authors.

## Ethics Statement

The studies involving human participants were reviewed and approved by the University Health Network - Research Ethics Board (Retrospective Study). Written informed consent for participation was not required for this study in accordance with the national legislation and the institutional requirements.

## Author Contributions

JL, SLA, and OM contributed to the conception and design of the work, acquisition/analysis/interpretation of the data; drafting of the work and revising it critically for important intellectual content; final approval of the version to be published and are in agreement to be accountable for all aspects of the work including the accuracy and integrity of the work. IP and SMA contributed to the acquisition and interpretation of the data; drafting of the work and revising it critically for important intellectual content; final approval of the version to be published and are in agreement to be accountable for all aspects of the work including the accuracy and integrity of the work. ZL and AL contributed to the analysis and interpretation of the data; drafting of the work and revising it critically for important intellectual content; final approval of the version to be published and is in agreement to be accountable for all aspects of the work including the accuracy and integrity of the work. JB, RT, JP, and KG-H contributed to the conception and design of the work, interpretation of the data; drafting of the work and revising it critically for important intellectual content; final approval of the version to be published and are in agreement to be accountable for all aspects of the work including the accuracy and integrity of the work. All authors contributed to the article and approved the submitted version.

## Conflict of Interest

The authors declare that the research was conducted in the absence of any commercial or financial relationships that could be construed as a potential conflict of interest.

## Publisher’s Note

All claims expressed in this article are solely those of the authors and do not necessarily represent those of their affiliated organizations, or those of the publisher, the editors and the reviewers. Any product that may be evaluated in this article, or claim that may be made by its manufacturer, is not guaranteed or endorsed by the publisher.
